# Are numerical scores important for grant proposals' evaluation? A cross sectional study

**DOI:** 10.12688/f1000research.139743.1

**Published:** 2023-09-26

**Authors:** Ivan Buljan, David G. Pina, Antonija Mijatović, Ana Marušić

**Affiliations:** 1 Department of Psychology, Faculty of Humanities and Social Sciences in Split, University of Split, Split, Croatia; 2 European Research Executive Agency, European Commission, Brussels, Belgium; 3 Department of Research in Biomedicine and Health and Center for Evidence-based Medicine, Medical School of Split, Split, Croatia

**Keywords:** Peer Review, Linguistic Characteristics, MSCA program

## Abstract

**Background:** In the evaluation of research proposals, reviewers are often required to provide their opinions using various forms of quantitative and qualitative criteria. In 2020, the European Commission removed, for the Marie Skłodowska-Curie Actions (MSCA) Innovative Training Networks (ITN) funding scheme, the numerical scores from the individual evaluations but retained them in the consensus report. This study aimed to assess whether there were any differences in reviewer comments’ linguistic characteristics after the numerical scoring was removed, compared to comments from 2019 when numerical scoring was still present.

**Methods:** This was an observational study and the data were collected for the Marie Skłodowska-Curie Actions (MSCA) Innovative Training Networks (ITN) evaluation reports from the calls of 2019 and 2020, for both individual and consensus comments and numerical scores about the quality of the research proposal on three evaluation criteria: Excellence, Impact and Implementation. All comments were analyzed using the Linguistic Inquiry and Word Count (LIWC) program.

**Results:** For both years, the comments for proposal’s strengths were written in a style that reflects objectivity, clout, and positive affect, while in weaknesses cold and objective style dominated, and that pattern remained stable across proposal status and research domains. Linguistic variables explained a very small proportion of the variance of the differences between 2019 and 2020 (McFadden R ^2^=0.03).

**Conclusions:** Removing the numerical scores was not associated with the differences in linguistic characteristics of the reviewer comments. Future studies should adopt a qualitative approach to assess whether there are conceptual changes in the content of the comments.

## Disclaimer

All views expressed in this study are strictly those of the authors and may in no circumstances be regarded as an official position of the European Research Executive Agency or the European Commission.

## Introduction

The process of evaluating research grant proposals has attracted considerable attention in the past decade. With the increasing amount of funding for research, there is a constant need for improvements in evaluation procedures for providing funding to the most promising project proposals. Recent scoping reviews on peer review for research funding recommends, among other propositions, that there is a need for the identification of interventions that are consistent in resolving peer review issues in proposal evaluation ( Recio-Saucedo *et al*., 2022; Shepherd *et al*., 2018). Studies on grant peer-review evaluation have mostly focused on the analysis of criteria used by expert reviewers when assessing proposals ( Abdoul *et al*., 2012; van Arensbergen and van den Besselaar, 2012; Hug and Aeschbach, 2020). Other studies have investigated the linguistic content of review reports ( van den Besselaar *et al*., 2018; Hren *et al.*, 2022). However, to the best of our knowledge, evidence is missing on how the requirement for numerically scoring a grant proposal, *i.e.* attributing a numerical/quantitative score to a proposal by a given reviewer, affects the way a reviewer comments or expresses opinions related to the proposal.

The evaluation process of grants submitted to EU research programs, the so-called Framework Programmes for research and innovation, consists usually of two consecutive steps, with each proposal going through (1) an individual evaluation, made by (typically three) different expert reviewers and (2) a consensus phase, where those reviewers agree on the final evaluation of the proposal. In both parts of the evaluation, the evaluation is normally focused on three criteria: a) research Excellence, b) Impact, and c) Implementation, for which comments must be given separately. Each criterion is attributed a score that determines the total score of the proposal. The result of the consensus stage is an evaluation summary report (ESR), consisting of the consolidated concerted opinions of the group of expert reviewers. Previous studies have established this approach as a stable procedure in the evaluation of research grant proposals ( Pina *et al*., 2021; Buljan *et al*., 2021).

In the previous Framework Programme, Horizon 2020 (H2020), some of the grant schemes faced changes in their scoring process. This was the case of the Marie Skłodowska-Curie Actions (MSCA), the flagship funding program dedicated to the promotion of researchers’ mobility and career development at all stages of their careers. In the past, expert reviewers were asked to provide comments and numerical scores for each of the three evaluation criteria in both their individual evaluations (the so-called Individual Evaluation Report – IER) and then at the level of the consensus, resulting in the final score of the evaluation summary report (ESR). For some of the funding schemes of MSCA, this approach was discontinued, and numerical scores were not attributed anymore at the level of individual evaluations (IER). Only textual comments were required at the IER stage, and numerical scores were used at the stage of the consensus for the ESR.

A recent study has indicated that proposal weaknesses have a greater effect on the ranking, compared to proposal strengths ( Hren *et al*., 2022). Based on this finding, the ranking of proposals would greatly depend on the reviewers’ ability to identify and describe the weaknesses. In cases when there is a large number of proposals, qualitative methods of analysis can be ineffective. Therefore, quantitative analyses of the text, *i.e.* tools that assess quantitative characteristics of the text, can be a solution, as they have been shown to be relevant for proposal evaluation ( Luo *et al*., 2022).

The objective of this study was to compare the linguistic characteristics of the comments related to the Excellence, Impact, and Implementation criteria in the evaluation reports of MSCA Innovative Training Networks (ITN) proposals submitted in 2019 and 2020, under H2020, in order to assess whether the removal of numerical scoring affected the structure of IER textual comments and whether this change was associated with the evaluation outcome at the consensus stage, *i.e.* ESR. We chose the ITN granting scheme, because it is, with around 1500 annual submissions and a success rate below 10%, among the most oversubscribed and competitive of the whole framework program.

## Methods

This study was preregistered on Open Science Framework: https://osf.io/t84ba.

### Ethics and consent

We worked on anonymized datasets, without insight about the actual content of the proposal, or the names of the applicants or experts evaluators, so that the regulations on personal data protection were not applicable.

### Study design

This was an observational, cross-sectional study conducted in 2022.

### Participants/sources of data

The data analyzed consisted in the IERs and the ESRs of all ITN proposals evaluated in the calls of 2019 and 2020. Each report includes textual comments referring to the different evaluation criteria. Scores of IER were only available for the year 2019. The anonymized quantitative data used for the analysis in this article is available on the Open Science Framework: https://osf.io/6bpvu/?view_only=.

### Assessment tool

Linguistic characteristics of experts’ comments were assessed using the Linguistic Inquiry and Word Count software ( Pennenbaker *et al*., 2015a, 2015b), a program that counts words related to different psychological states and phenomena and gives a score that is a proportion of the specific category in the entire text.

### Variables analyzed

We collected the data on the proposal status after evaluation (“Main list”, “Reserve list” or “Rejected”), call in which they were submitted (2019 or 2020), research area, total evaluation scores, as well as numerical scores for Excellence, Implementation and Impact criteria, together with corresponding comments which separately described proposal strengths and weaknesses. We separately analysed IERs and ESRs.

For the evaluation purposes, proposals are categorized in eight panels: Economics-ECO, Social sciences-SOC, Mathematics-MAT, Physics-PHY, Chemistry-CHE, Engineering-ENG, Environmental sciences-ENV, Life sciences-LIF. For this study, we clustered the MAT-PHY-CHE-ENG-ENV into one single “research domain”: PHYENG. So, the three research domains in this study were PHYENG, ECOSOC and LIF. 

LIWC variables were calculated separately for strengths and weaknesses for each of the criteria assessed: They included the word count and the text tone of the evaluation report ( Kaatz *et al*., 2015; Kacewicz *et al*., 2014; Pennebaker *et al*., 2015a):

a) Analytical tone: higher scores indicate the logical and hierarchical style of writing;
b) Clout tone: higher scores indicate confidence or leadership, with lower scores indicating insecure writing;
c) Authenticity: high scores indicate writing honestly and humbly, with expressing views as personal opinion;
d) Emotional tone: higher scores on emotional tone indicate a higher proportion of words related to a more positive emotional tone.

### Bias

To eliminate potential sampling bias, we collected data for the whole cohort of submitted MSCA ITN proposals in 2019 and 2020.

### Statistical analysis

The descriptive data was presented as frequencies and percentages for project status and research domain. Text characteristics were presented as means and standard deviations, or as means and 95% confidence intervals in cases of figures. We first compared the differences on all variables using a t-test or Chi squared, depending on the nature of the variables. A P value threshold of less than 0.001 was considered to be significant in t test. Variables which were not significant were excluded from further analysis. We used logistic regression to compare differences between the two call years, in which proposal variables (proposal status, word count for research excellence weaknesses, word count for implementation strengths, and negative affect levels for implementation strengths) were predictors and the year of the call was the criterion. The level of significance was set to 0.05. The analysis was done using the R statistical program ( R Core Team, 2021) and the JAMOVI package for statistical analysis ( The jamovi project, 2023).

## Results

The number of evaluated proposals was similar in 2019 (n=1554) and 2020 (n=1503). The number of rejected proposals was 1367 (87.9%) for 2019 and 1333 (88.6%) for 2020, 128 (8.2%) and 148 (9.8%) funded proposals, and 59 (3.8%) and 22 (1.5%) proposals on the reserve list in 2019 and 2020, respectively. The majority of the proposals was from physical sciences and engineering (n=1004, 64.6% for 2019 and n=947, 63.0 for 2020), followed by life sciences (n=391, 25.1% for 2019 and n=387, 25.8%) and economics and social sciences (n=159, 10.2% for 2019 and n=169, 11.2% for 2020).

**Figure 1.**
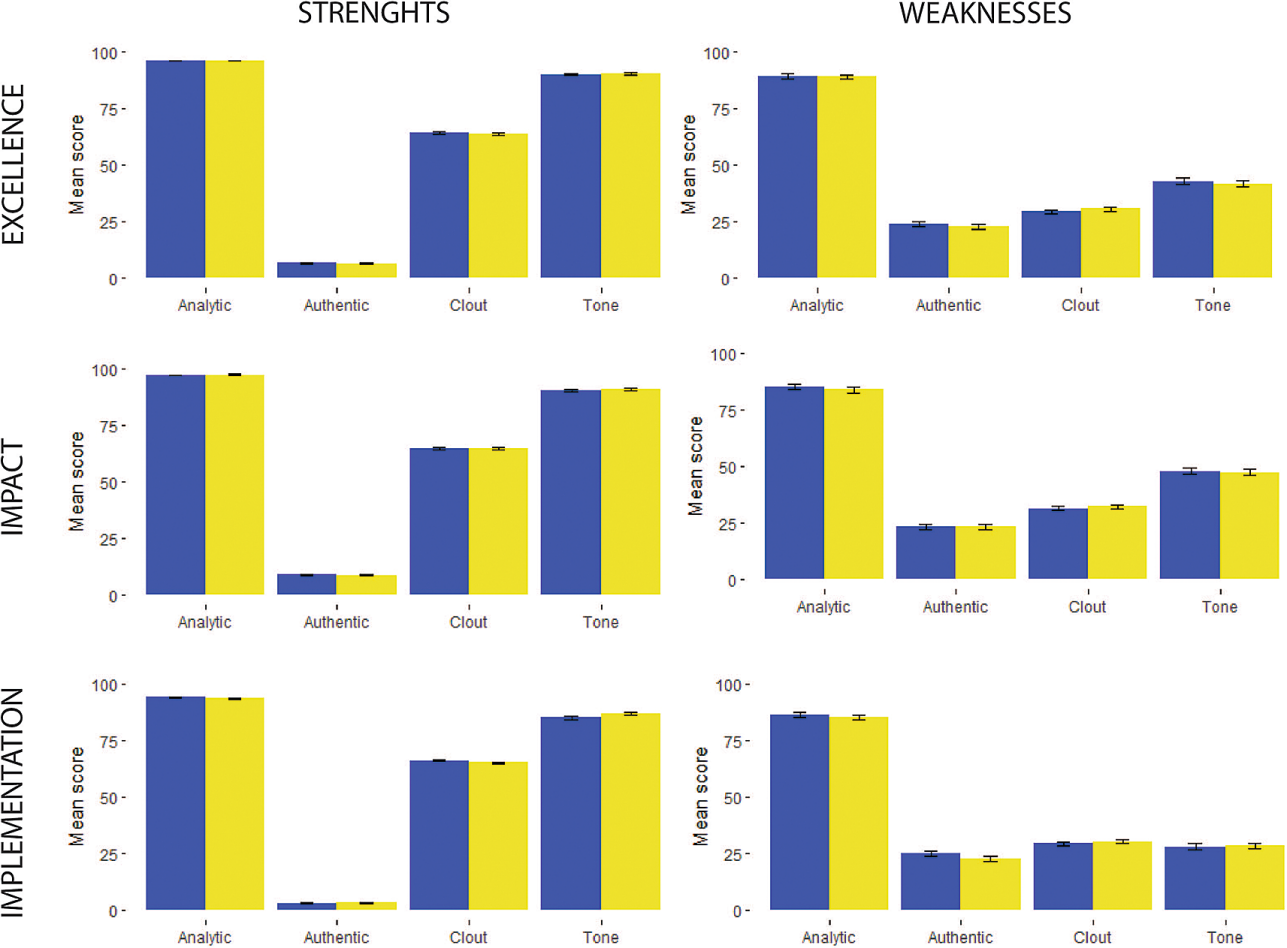
Linguistic characteristics scores of consensus report reviewer’s comments about proposal between 2019-blue and 2020-yellow.

**Figure 2.**
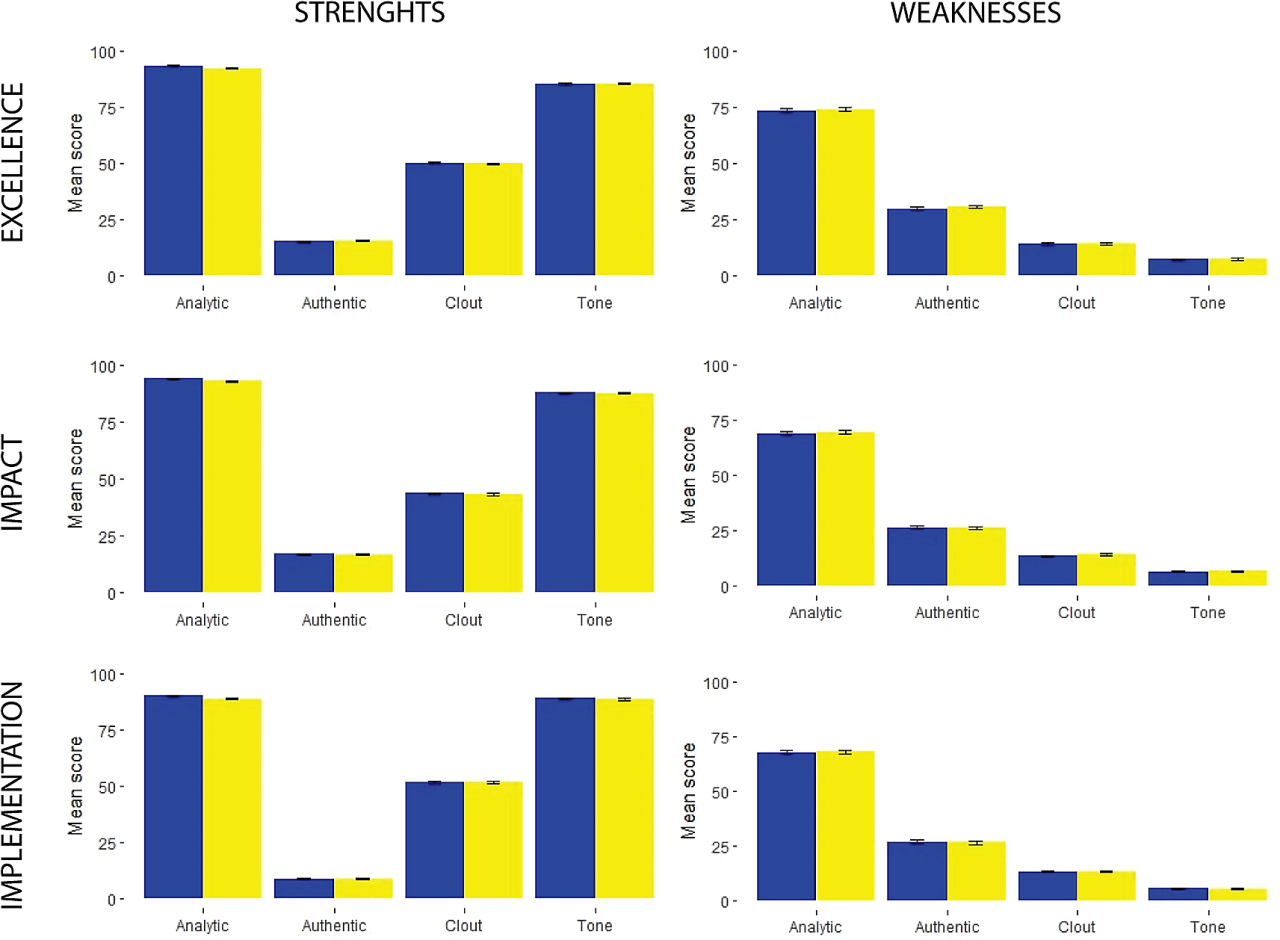
Linguistic characteristics scores of individual evaluation report reviewer’s comments about proposal between 2019-blue and 2020-yellow.

Overall, review comments were written predominantly in an analytic and objective language, which was indicated by the high level of Analytical tone and low levels of Authenticity; this indicates that a small proportion of reviewers formulated their arguments as personal opinions, rather than objective comments ( Figures 1 and 2).

There was a greater presence of clout and emotional tone in the description of the proposal’s strengths ( Figures 1 and 2). Also, a greater proportion of the comments describing the strengths of the proposal had more words related to the positive emotional tone ( Figures 1 and 2).

The acceptance of a proposal was predicted by the linguistic characteristics of the comments related to the weaknesses in the proposal, more specifically lower analytical tone across all three criteria’s weaknesses, a higher negative emotional tone for research excellence and impact weaknesses, and higher clout in research excellence ( Table 1). In total, those predictors explained around 30% of the variance of the criteria (McFadden R ^2^=0.30). On the other hand, the differences between 2019 and 2020 were negligible, explaining around 3% of the variance (McFadden R ^2^=0.03) ( Table 2). These predictors included the number of words in excellence strengths (both for individual reviewers and consensus reports), lower analytical tone for excellence and impact in individual reports and higher emotional tone in consensus reports.

**Table 1.** Ordinal logistic regression model for prediction of proposal status by linguistic characteristics of reviewer’s comments ^a^.

Variable	Odds ratio (95% CI)	P Value
IER WC weaknesses excellence	0.98 (0.98 to 0.99)	<0.001
IER Analytic weaknesses excellence	0.99 (0.99 to 0.99)	0.010
CR WC weaknesses excellence	0.99 (0.98 to 0.99)	<0.001
CR Analytic weaknesses excellence	0.97 (0.96 to 0.98)	<0.001
CR Clout weaknesses excellence	1.02 (1.01 to 1.02)	<0.001
CR Authentic weaknesses excellence	1.01 (1.00 to 1.01)	0.008
CR Analytic weaknesses impact	0.98 (0.98 to 0.99)	<0.001
CR Clout strengths implementation	0.95 (0.93 to 0.97)	<0.001
CR Analytic weaknesses implementation	0.98 (0.98 to 0.99)	<0.001

CI - Confidence interval; IER - Individual Evaluation Report; CR - Consensus Report; WC - Word Count.

^a^ The categories were ordered as following: rejected, reserved, main listed. Higher odds ratio indicates greater probability for acceptance.

**Table 2.** Logistic regression in predicting ITN calls with individual (IER) and consensus (CR) comment characteristics ^a^.

Variable	Odds ratio (95% CI)	P Value
IER WC strengths excellence	1.01 (1.00 to 1.01)	<0.001
IER Analytic strengths excellence	0.98 (0.96 to 0.99)	0.002
IER Analytic strengths impact	0.98 (0.97 to 0.99)	0.007
IER Clout weaknesses impact	1.01 (1.00 to 1.02)	0.023
CR WC weaknesses excellence	1.01 (1.00 to 1.01)	<0.001
CR WC strengths implementation	1.01 (1.00 to 1.02)	<0.001
CR Emotional tone strengths implementation	1.01 (1.01 to 1.01)	0.003

CI - Confidence interval; IER - Individual Evaluation Report; CR - Consensus Report; WC - Word Count.

^a^ Criterion variable was call year: the 2019 call was labeled as 0 and the 2020 call as 1.

With regard to the differences in textual characteristics between consensus report evaluation and individual evaluation scores between 2019 and 2020, the observed pattern of greatest differences between consensus score and individual scores in emotional tone was stable across different proposal status ( Table 3) and research domains ( Table 4). Emotional tone was overall greater for consensus score results.

**Table 3.** Average scores (mean, standard deviation) for differences between consensus score and individual evaluation scores across different outcome status categories and between 2019 and 2020 ^a^.

	Main list		Reserve list		Rejected list	
	2019 (n=128)	2020 (n=148)	2019 (n=59)	2020 (n=22)	2019 (n=1367)	2020 (n=1333)
**Excellence – strengths:**						
Word count	-57.1 (101.8)	-64.4. (-111.7)	-46.3 (99.0)	-63.0 (87.8)	-7.8 (104.8)	-11.0 (105.2)
Analytic tone	3.1 (5.8)	4.2 (6.8)	2.8 (3.9)	1.7 (5.1)	2.6 (7.0)	3.8 (7.6)
Clout tone	14.1 (7.8)	14.7 (9.2)	14.3 (9.1)	10.2 (9.3)	13.9 (9.8)	13.9 (9.7)
Authentic tone	-9.4 (7.6)	-9.8 (8.3)	-8.5 (8.9)	-9.4 (6.6)	-8.4 (9.3)	-9.0 (9.6)
Emotional tone	6.8 (13.9)	5.1 (13.6)	3.9 (12.2)	7.5 (12.3)	4.9 (14.4)	4.9 (15.1)
**Excellence – weaknesses:**						
Word count	-20.6 (59.0)	-2.5 (85.4)	-23.4 (51.8)	-25.5 (25.8)	-5.9 (59.1)	3.5 (67.3)
Analytic tone	3.8 (36.7)	8.7 (36.2)	13.3 (29.4)	7.5 (27.5)	17.1 (19.4)	16.2 (19.0)
Clout tone	5.0 (24.8)	11.2 (26.9)	13.4 (21.8)	10.8 (20.6)	16.1 (17.0)	16.9 (16.5)
Authentic tone	5.2 (30.8)	-0.5 (29.4)	4.0 (31.6)	2.6 (23.3)	-7.3 (22.9)	-8.9 (22.7)
Emotional tone	28.2 (24.2)	32.8 (27.2)	34.7 (26.2)	29.1 (23.6)	36.3 (25.4)	34.6 (25.4)
**Impact – strengths:**						
Word count	-55.9 (102.4)	-45.8 (95.0)	-39.6 (94.2)	-31.0 (90.1)	-0.3 (94.5)	-4.2 (91.0)
Analytic tone	1.8 (12.7)	4.1 (8.9)	1.6 (4.7)	-2.6 (21.1)	3.3 (7.3)	4.5 (9.0)
Clout tone	20.5 (12.0)	20.2 (9.8)	19.0 (10.1)	16.2 (19.2)	21.5 (11.0)	21.6 (11.7)
Authentic tone	-8.5 (9.1)	-8.0 (11.3)	-7.7 (9.5)	-8.9 (11.9)	-8.0 (11.6)	-8.0 (11.5)
Emotional tone	5.0 (15.2)	7.1 (11.9)	5.4 (11.4)	-3.2 (16.5)	2.4 (14.2)	3.1 (14.3)
**Impact – weaknesses:**						
Word count	12.3 (46.9)	-6.2 (60.8)	-18.5 (38.6)	-21.4 (22.2)	-6.7 (43.9)	0.3 (52.2)
Analytic tone	5.6 (33.2)	-0.6 (39.6)	10.1 (37.2)	5.2 (37.7)	17.4 (24.0)	16.1 (24.4)
Clout tone	4.8 (26.7)	5.8 (28.7)	10.1 (23.8)	13.7 (31.8)	19.6 (20.9)	19.3 (21.0)
Authentic tone	5.0 (28.7)	4.8 (26.6)	5.1 (26.9)	2.8 (22.2)	-4.4 (25.1)	-4.1 (24.5)
Emotional tone	32.3 (27.0)	31.0 (25.0)	39.1 (31.6)	34.6 (27.5)	42.4 (28.2)	41.8 (28.5)
**Implementation – strengths:**						
Word count	1.3 (66.6)	6.0 (77.7)	9.9 (47.0)	16.3 (42.5)	-3.2 (54.4)	2.6 (61.6)
Analytic tone	3.6 (7.8)	4.7 (11.7)	5.5 (7.9)	-0.2 (10.7)	4.0 (7.5)	4.8 (8.9)
Clout tone	13.8 (7.5)	12.9 (10.3)	13.4 (8.6)	11.3 (5.8)	14.3 (9.1)	13.3 (9.8)
Authentic tone	-6.4 (4.6)	-6.2 (6.0)	-5.7 (5.0)	-5.1 (4.6)	-5.5 (5.3)	-5.4 (6.0)
Emotional tone	-0.5 (14.5)	-0.7 (15.6)	-5.7 (15.7)	-2.3 (14.9)	-4.1 (17.4)	-2.0 (16.1)
**Implementation – weaknesses:**						
Word count	-9.7 (47.8)	-0.7 (59.4)	-15.8 (48.5)	-5.0 (23.3)	0.5 (50.3)	6.4 (52.9)
Analytic tone	17.1 (36.3)	6.3 (37.4)	16.8 (29.3)	12.8 (35.3)	18.8 (23.0)	18.4 (23.4)
Clout tone	10.3 (23.2)	7.7 (24.1)	12.9 (22.4)	10.7 (23.2)	16.8 (18.7)	17.9 (18.5)
Authentic tone	9.3 (28.8)	1.6 (26.9)	9.0 (31.2)	5.5 (33.0)	-3.3 (25.1)	-4.5 (24.3)
Emotional tone	24.6 (27.4)	26.7 (25.4)	24.3 (29.5)	21.4 (24.1)	22.4 (26.1)	22.8 (26.4)

^a^ The difference was calculated for each project as the difference between the Consensus score result and the Individual evaluation score.

**Table 4.** Average scores for differences between consensus score and individual evaluation scores across different research domains and between 2019 and 2020 ^a^.

	Research domain (M, SD)					
	ECO/SOC		LIF		PHY/ENG	
	2019 (n=159)	2020 (n=169)	2019 (n=391)	2020 (n=387)	2019 (n=1004)	2020 (n=947)
Excellence strengths						
Word count research	-16.6 (101.1)	-21.3 (111.5)	-5.5 (108.2)	-19.5 (109.0)	-15.8 (104.9)	-15.2 (105.3)
Analytic tone research	1.1 (5.3)	1.9 (4.1)	3.4 (9.1)	3.4 (5.8)	2.6 (5.9)	4.3 (8.4)
Clout tone research	15.9 (8.6)	13.9 (9.6)	14.7 (9.8)	13.9 (9.3)	13.4 (9.7)	13.9 (9.8)
Authentic tone research	-10.1 (8.3)	-11.7 (10.8)	-9.0 (8.5)	-9.2 (9.9)	-8.1 (9.4)	-8.5 (8.9)
Emotional tone research	8.1 (13.4)	6.3 (15.7)	3.4 (14.4)	4.5 (14.9)	5.1 (14.4)	4.5 (14.9)
Excellence weaknesses						
Word count research	8.8 (85.6)	0.6 (68.9)	-0.4 (58.4)	15.7 (73.8)	-13.2 (53.1)	-2.6 (66.1)
Analytic tone research	12.8 (21.0)	16.5 (19.9)	15.8 (21.3)	15.5 (22.7)	16.4 (22.5)	15.0 (22.7)
Clout tone research	18.4 (19.3)	19.3 (18.2)	15.7 (16.9)	16.8 (16.4)	14.3 (18.5)	15.4 (18.4)
Authentic tone research	-8.8 (23.9)	-13.7 (22.6)	-6.1 (23.5)	-8.0 (23.2)	-5.2 (24.7)	-6.8 (23.7)
Emotional tone research	33.3 (24.6)	28.4 (23.0)	38.4 (25.8)	35.7 (25.8)	34.9 (25.3)	34.9 (25.8)
Impact strengths						
Word count	-11.0 (89.5)	-11.9 (97.0)	-1.4 (96.6)	-3.7 (92.3)	7.6 (97.6)	-10.2 (91.3)
Analytic tone	1.7 (7.1)	3.2 (11.2)	4.4 (7.5)	5.2 (8.3)	2.8 (7.9)	4.2 (9.2)
Clout tone	23.6 (11.8)	21.5 (13.1)	21.3 (11.1)	21.2 (11.8)	20.9 (20.9)	21.4 (11.4)
Authentic tone	-7.8 (11.4)	-9.0 (12.1)	-7.7 (11.0)	-7.5 (13.1)	-8.2 (11.5)	-8.1 (10.7)
Emotional tone	5.3 (13.3)	7.0 (12.7)	1.0 (13.9)	2.1 (12.7)	3.0 (14.4)	3.3 (14.8)
Impact weaknesses						
Word count	8.5 (68.6)	2.5 (55.8)	-6.5 (40.3)	5.5 (58.3)	-10.6 (39.8)	-3.7 (49.7)
Analytic tone	12.0 (22.4)	16.6 (18.2)	16.8 (25.9)	13.1 (30.0)	16.5 (26.1)	14.3 (27.0)
Clout tone	21.5 (19.8)	20.7 (18.8)	20.3 (22.5)	19.0 (23.1)	16.6 (22.0)	16.9 (22.7)
Authentic tone	-4.2 (22.8)	-10.5 (19.6)	-4.8 (25.7)	-2.2 (24.1)	-2.6 (26.0)	-2.2 (25.8)
Emotional tone	42.3 (26.3)	39.0 (25.5)	42.7 (29.8)	42.0 (27.4)	40.9 (28.1)	40.4 (29.2)
Implementation strengths						
Word count	1.3 (71.5)	15.9 (68.2)	3.8 (56.4)	6.4 (63.0)	-5.2 (51.7)	-0.5 (61.9)
Analytic tone	3.7 (6.8)	3.9 (8.8)	5.1 (8.4)	4.2 (10.0)	3.7 (7.2)	5.1 (9.1)
Clout tone	14.2 (9.2)	11.9 (10.1)	14.7 (9.1)	13.9 (10.5)	14.0 (8.9)	13.2 (9.3)
Authentic tone	-5.5 (4.9)	-5.7 (4.5)	-5.6 (5.1)	-6.0 (6.8)	-5.6 (5.4)	-5.2 (5.8)
Emotional tone	-7.2 (19.3)	-1.4 (15.3)	-5.5 (15.9)	-4.5 (16.4)	-2.6 (17.1)	-0.9 (15.9)
Implementation weaknesses						
Word count	6.4 (68.7)	1.1 (54.7)	2.2 (48.7)	13.2 (56.8)	-3.4 (47.0)	3.2 (51.4)
Analytic tone	13.5 (26.7)	17.8 (28.0)	19.8 (23.6)	17.7 (26.4)	18.9 (24.6)	16.8 (24.7)
Clout tone	17.7 (22.6)	19.1 (19.1)	18.1 (19.0)	18.7 (18.2)	15.0 (18.9)	15.6 (20.0)
Authentic tone	-3.2 (22.6)	-6.8 (23.3)	-5.9 (25.6)	-5.9 (24.3)	0.1 (26.49	-2.3 (25.2)
Emotional tone	31.4 (28.1)	25.7 (27.3)	24.5 (27.7)	23.8 (27.3)	20.5 (25.2)	22.4 (25.9)

ECO/SOC - Economic and social sciences; LIF - Life sciences; PHY/ENG - Physics and engineering.

^a^ The difference was calculated as Consensus score result-Individual evaluation score result; for every corresponding project.

## Discussion

In this study, which included all ITN proposals from 2019 and 2020 calls, we aimed to assess whether the changes in the evaluation procedure were related to differences in characteristics of review reports. We found that the differences in linguistic characteristics between reports fromboth calls (2019 and 2020) were small and negligible from a practical point, indicating that the removal of numerical scores did not result in meaningful changes in the reports’ comments, assessed by quantitative text analysis. For both calls, the comments were written objectively, with weaknesses written with less emotion and more analytically than the proposals’ strengths. On the other hand, we found that the final status of the proposals ( *i.e.* main-listed or rejected) can be predicted by the linguistic characteristics of the reviewer’s comments, especially the tone related to the identified weaknesses, indicating that weaknesses may be crucial in proposal evaluation.

The comments’ text was written mostly using formal language, indicated by high levels of analytical tone, both for strengths and weaknesses. The same feature was observed in a previous study performed on journals’ reports from peer reviewers ( Buljan *et al.*, 2021). Our results also provide evidence for a general advice to the applicants – to be very focused on the objective structure of their proposal ( Baumert *et al*., 2022). When emphasizing proposal strengths, due to the low levels of authenticity, the reviewers less frequently used personal pronouns like “I” or “we”, probably to present the proposal strengths as factual information, and not as a personal opinion. This finding is contrary to the study of Thelwall *et al*. (2023), which pointed out that higher use of first pronouns in reviews is related with higher proposal quality. On the other hand, in the description of weaknesses, the reviewers more often presented the information as their personal opinion. This finding is further supported by high levels of clout tone in the description of strengths. The clout – the tone which indicates writing from a position of power – was much higher in the description of the project strengths than in the description of the weaknesses, from which we can conclude that reviewers were more certain in their evaluation. The emotional tone was more positive in the description of strengths, probably because of the use of words related to the project’s probable success. There was a greater presence of clout and emotional tone in the description of the proposal’s strengths, which indicates that reviewers wrote with less confidence when discussing the potential flaws of the proposals, compared to when they mentioned the proposal’s strengths. In that respect, it is to be noted that the EC services instruct reviewers that evaluation reports should not express opinions, but rather evaluate factual elements of the proposals.

The principal difference between consensus and individual scores was in the emotional tone score. Across different categories, the emotional tone was higher in consensus ratings rather than in individual evaluation score, indicating more positive tone of the outcome of the consensus process. This may be due to the fact that only the concensus report is sent our to applicants. The IER remains an internal (intermediate) report and is not externalised and sent to applicants. In a previous study, we found that the agreement between reviewers was very high ( Pina *et al*., 2021). At the time of the individual evaluation, the reviewers do not know whether other reviewers will agree with them. It is possible that, when reviewers need to write a consensus evaluation, they are not limited by the objective language in the evaluation of the proposal, since it is established that other reviewers agree with their opinion, so the tone is more relaxed and positive.

Our previous study of the predictive value of comments on proposals’ strengths and weaknesses in ITN evaluation process used both qualitative and quantitative (machine learning) approach ( Hren *et al*., 2022). Our present results partially confirm the results of that study, which found that proposals’ weaknesses are more predictive of its evaluation outcome ( Hren *et al*., 2022). However, we found that only some elements in the weaknesses are predictive of the proposal status. Specifically, a higher analytical tone and fewer negative evaluation words in comments related to proposals’ weaknesses were associated with a more favorable funding outcome . It needs to be noted that themes that served as predictors in the regression model were identified qualitatively and were better predictors (explained around 55% of the criteria) compared to our quantitative text analysis (around 30% of the criteria). However, due to the large number of proposals, linguistic characteristics of reviewers’ comments may serve as an additional tool in the proposed evaluation, as advised by others ( Luo *et al*., 2022).

The finding that we did not observe meaningful differences in tone of reviewers’ comments needs to be interpreted in the light of several limitations. It should be noted that our entire quantitative text analysis process was made by dictionary-based text analysis algorithms, which may slightly deviate from manual analysis ( Luo *et al*., 2022), being still predictive for proposal funding outcomes, and that was partially reproduced in our study. One aspect of quantitative text analysis, sentiment analysis or analysis of the text tone, could serve as a useful tool to determine whether there were any differences in the evaluation performed by reviewers after the removal of individual numerical scores, as reviewers were a common part of both procedures. We only focused on the linguistic characteristics of the comments related to the positive and negative sides of the proposals. Qualitative analysis of the proposals would give input on the potential differences between the two calls but, due to the number of the proposals, there is a question of practical value for such an approach. We do not have information about who the reviewers were, which may present relevant information since some individual characteristics, such as experience in research or reviewing, may influence the review process ( Seeber *et al*., 2021). Based on our evaluation, we found no evidence that the removal of numerical scoring produced any differences in the evaluation output.

## Conclusions

This study assessed whether removing of numerical scores will have a significant effect on the evaluation procedure. The findings indicate that the removal of numerical scores did not contribute to meaningful differences in the evaluation procedure of H2020 ITN proposals or its outcome. Those results support the finding that the procedure used for the evaluation of MSCA grant proposals is very robust and stable.

## Author contributions

**Ivan Buljan**, Conceptualization, Data Curation, Formal Analysis, Investigation, Methodology, Project Administration, Visualization, Writing – Original Draft Preparation, Writing – Review & Editing; **David G Pina**, Conceptualization, Data Curation, Investigation, Methodology, Project Administration, Resources, Writing – Original Draft Preparation, Writing – Review & Editing; **Antonija Mijatović,** Conceptualization, Data Curation, Formal Analysis, Investigation, Methodology, Writing – Original Draft Preparation, Writing – Review & Editing and **Ana Marušić**, Conceptualization, Data Curation, Formal Analysis, Funding Acquisition, Investigation, Methodology, Project Administration, Resources, Supervision, Visualization, Writing – Original Draft Preparation, Writing – Review & Editing.

## Data Availability

Open Science Framework: Are numerical scores important for grant evaluation?, https://osf.io/6bpvu/?view_only= ( Buljan *et al*., 2021). This project contains the following underlying data:
- Data file 1. Dataset_anonymized.csv Open Science Framework: Are numerical scores important for grant evaluation?, https://osf.io/6bpvu/?view_only= ( Buljan *et al*., 2021). This project contains the following extended data:
- Extended data.docx Open Science Framework: STROBE checklist for “Are numerical scores important for grant evaluation? A cross sectional study”, https://osf.io/6bpvu/?view_only= ( Buljan *et al*., 2021). Data are available under the terms of the Creative Commons Zero “No rights reserved” data waiver (CC0 1.0 Public domain dedication).
