# Supplementary material for: Are numerical scores important for grant assessment? A cross-sectional study
Source: F1000Res. 2024 Sep 5;12:1216. Originally published 2023 Sep 26. [Version 2] doi: 10.12688/f1000research.139743.2 (PMC11362741; doi:10.12688/f1000research.139743.2)
Supplement: Peer review history

# Reviewer report: Reviewer response for version 2

**DOI:** 10.5256/f1000research.170996.r320980

**Published:** 2024-09-11

**Authors:** Seba Qussini, Dr. Samer Hammoudeh

**Affiliations:** 1 Medical Research Center, Hamad Medical Corporation, Doha, Qatar; 2 Center for Biomedical Ethics and Law, KU Leuven (Ringgold ID: 26657), Leuven, Flanders, Belgium; 3 Hamad Medical Corporation Medical Research Center, Doha, Doha, Qatar

We thank the authors for implementing the necessary revisions.

Is the work clearly and accurately presented and does it cite the current literature?

Yes

If applicable, is the statistical analysis and its interpretation appropriate?

Yes

Are all the source data underlying the results available to ensure full reproducibility?

Yes

Is the study design appropriate and is the work technically sound?

Yes

Are the conclusions drawn adequately supported by the results?

Yes

Are sufficient details of methods and analysis provided to allow replication by others?

Yes

Reviewer Expertise:

Research Integrity and Peer review

We confirm that we have read this submission and believe that we have an appropriate level of expertise to confirm that it is of an acceptable scientific standard.

# Reviewer report: Reviewer response for version 1

**DOI:** 10.5256/f1000research.153043.r246534

**Published:** 2024-08-29

**Authors:** Jan-Ole Hesselberg

**Affiliations:** 1 Department of Psychology, University of Oslo, Oslo, Norway

**GENERAL COMMENT**

 This is an interesting and well-written article. I have few specific comments and have no substantial comments regarding the conclusions. 

 My main concern are the deviations from the preregistration, both regarding the measured variables and the statistical analyses. These deviations seem to be significant and increase the risk of bias. I want to stress I believe the methods used are sound and that there are likely good reasons for the deviations. However, the reader should be made aware of them and the authors should comment on:

 1) Which changes were made to the methods

 2) Why they were made

 3) If the results would have been different if methods described in the preregistration had been followed

 I would prefer if the Methods section had contained a subsection called "Differences between preregistration and article" and that the results section contained comments regarding what the results would have been if the preplanned analyses had been done. 

 To help the reader of this review to see the differences. Here are the **preplanned measured variables **described in the preregistration ****(https://osf.io/t84ba):

 "The outcome variable will be the difference in final scores between 2019 and 2020. Input variables will be: year of the calls; evaluation panel; country of the coordinator (high and low research performing countries); linguistic characteristics of the individual expert evaluation in each of the three evaluation criteria (Excellence, Impact, Implementation) – LIWC analysis and another measure of sentiment analysis like RSentiment."

 There are no mentions of calculated indices or transformations

 And here are the **preplanned statistical analyses** described in the preregistration ****(https://osf.io/t84ba):

*"Statistical models*

 The categorical data will be presented as frequencies and percentages, while numerical data will be presented as means and standard deviations in the descriptive part and with 95% confidence intervals in the inferential part of the analysis. We will compare the differences in linguistic characteristics between 2019 and 2020 and simultaneously compare linguistic characteristics between different reviewer decisions using two-way ANOVA. The data in the models will be presented as group means and 95% confidence intervals while final results will be expressed with squared eta effect size. 

*Transformations*

 None planned.

*Inference criteria*

 We will use the standard p<.05 criteria for determining if the ANOVA and the post hoc test suggest that the results are significantly different from those expected if the null hypothesis were correct. The post-hoc Tukey-Kramer test adjusts for multiple comparisons."

**SPECIFIC COMMENTS**

*Introduction*

 "Previous studies have established this approach as a stable procedure in the evaluation of research grant proposals"

 What is meant by "stable procedure" in this quote? 

*Participants/sources of data*

 Thank you for sharing the data. It would be very helpful if you could also share the analytic code (the R script).

*Variables analyzed*

 Please comment on the changes from the preregistration. Among other things, it seems like "country of coordinator" was left out and that the clustering of PHYENG and the use of "proposal status after evaluation" was not preplanned.

*Statistical analysis*

 Please comment on the changes from the preregistration. 

*Discussion*

 The phrase "can be predicted" is used. I believe it would be helpful to add some wording to describe to the reader to what *extent* it can be predicted.

Is the work clearly and accurately presented and does it cite the current literature?

Yes

If applicable, is the statistical analysis and its interpretation appropriate?

Partly

Are all the source data underlying the results available to ensure full reproducibility?

Partly

Is the study design appropriate and is the work technically sound?

Partly

Are the conclusions drawn adequately supported by the results?

Yes

Are sufficient details of methods and analysis provided to allow replication by others?

Partly

Reviewer Expertise:

Judgement and decision-making, peer-review, inter-rater agreement

I confirm that I have read this submission and believe that I have an appropriate level of expertise to confirm that it is of an acceptable scientific standard, however I have significant reservations, as outlined above.

# Response

**Published:** 2024-09-02

**Authors:** Ivan Buljan

**Affiliations:** Department of Psychology, Faculty of Humanities and Social Sciences in Split, Split, Croatia

Dear reviewer, 

 Thank you for your insightful suggestions to enhance our article. We have incorporated a new section titled "Deviations from the Protocol" and provided a detailed description of the revised methods in the latest version. The new version was submitted last week and will be available in the coming weeks. If you feel the changes still fall short, we are open to making further adjustments.

 Kind regards, 

 Ivan Buljan

# Reviewer report: Reviewer response for version 1

**DOI:** 10.5256/f1000research.153043.r246528

**Published:** 2024-03-14

**Authors:** Seba Qussini, Dr. Samer Hammoudeh

**Affiliations:** 1 Medical Research Center, Hamad Medical Corporation, Doha, Qatar; 2 Center for Biomedical Ethics and Law, KU Leuven (Ringgold ID: 26657), Leuven, Flanders, Belgium; 3 Hamad Medical Corporation Medical Research Center, Doha, Doha, Qatar

The study in hand evaluates whether reviewer comments on research proposals differed after the restructuring of the Marie Sklodowska-Curie Actions (MSCA) Innovative Training Networks (ITN) scoring scheme by eliminating the possibility of scoring using numbers. The authors utilize proposal requests data from 2019 and 2020 and found that linguistic characteristics did not differ after the removal of numerical scores from the specified scoring scheme. The following comments are applicable to the current draft of the manuscript:

- It would be strongly advised that the authors run grammar and typo check, or to obtain language and editing services. The current version of the manuscript has multiple occasions of misspelled words, grammatical errors, and typos. Some examples are figure 1 or 2: the word strengths. First paragraph of discussion: from both . Third paragraph of discussion: the word consensus.
- Please add an abbreviation section at the end of the manuscript, and make sure that all abbreviations are spelled out at the first encounter throughout the manuscript.
- The current study fills a current gap in the literature as identified by the authors early in the introduction section. This section can probably be restructured for better understanding of the readers, in the following manner: ideally, the first paragraph would discuss general aspects of peer review, therefore, the gap in literature can be moved towards the end of the introduction, after discussing a few aspects, models, shortcomings, etc.
- Additionally, the authors may provide examples for the quantitative characteristics in the introduction, and/or further elaborate on the effect of quantitative characteristics on reviewers’ evaluation. Providing more information regarding the Marie Skłodowska-Curie Actions (MSCA) would probably help supplement this section and provide a better understanding of the dynamics of this grant. Furthermore, it could have been useful to touch on other possible proposals’ evaluating methods such as scoring and ranking, as can be found in[1]
- The last paragraph may be used to highlight further the gap in the literature that the authors aim to bridge with this project, indicating that peer review is highly versatile and a diverse process, yet further focusing on the need to address the general lack of theoretical understanding in multiple aspects of peer review, as can be seen in:  [2], and/or the paucity of experimental research in proposals’ peer review as summarized by [3]
- The authors may want to further elaborate on the reasons for removing the numerical score from MSCA? Was it for the effect of simplifying the peer review process on reviewers’ engagement and quality of report? as pointed out by *[4]*
- Registering with the Open Science Framework is a plus.
- The study design is cross sectional but not observational. The latter terminology may be removed.
- What is the justification for including data from only 2019 and 2020? Why not expand on the size of data by including more years.
- Further information may be added to the assessment tool, Linguistic Inquiry and Word Count (LWIC) software, such as the validity and reliability, the LWIC scores, possible cutoffs, totals, etc. The authors do provide a reference, but it would just be easier to have it readily accessible while reading.
- Under the bias heading: What is the role of including the whole cohort of submitted proposals in 2019 and 2020 in eliminating potential sampling bias? Please make it clear for the readers.

- In the statistical analysis section: How were the predictors established? Was it based on an analysis or from the literature? It would be advised to clarify this detail.
- Additionally, please provide justification for using the specified p-value 0.001 in your model, as it appears too stringent.
- In the results section: The majority of proposals were from the physical science and engineering fields. Did this play a role in the outcome of the study, where no differences were detected before and after? The authors may want to expand on this in the discussion section.
- Additionally, is it possible that COVID19 resultant urgency have affected the review of proposals in 2020?
- It would also be useful to further elaborate on the blinding model adopted by the MSCA and how they may have influenced the results.
- In table 3: How can the average word count be negative? Please provide an explanation.
- It would be great to supplement the discussion section with a few recommendations for future researchers into the topic.
- Have the authors considered adjusting for possible confounding factors, such as research domain, etc.? If not , please discuss the possible effects of such confounding factors in the Discussion section.

Overall, the authors follow a logical order in introducing the main aspects of the study, provide sufficient details in some areas and are lacking minute details in other areas. The findings are clearly conveyed along with a proper interpretation of the results. The value of such work would probably benefit research administrators along with policy makers.

Is the work clearly and accurately presented and does it cite the current literature?

Yes

If applicable, is the statistical analysis and its interpretation appropriate?

Yes

Are all the source data underlying the results available to ensure full reproducibility?

Yes

Is the study design appropriate and is the work technically sound?

Yes

Are the conclusions drawn adequately supported by the results?

Yes

Are sufficient details of methods and analysis provided to allow replication by others?

Yes

Reviewer Expertise:

Research Integrity and Peer review

We confirm that we have read this submission and believe that we have an appropriate level of expertise to confirm that it is of an acceptable scientific standard, however we have significant reservations, as outlined above.

## References

[1] : A new approach to grant review assessments: score, then rank. *Res Integr Peer Rev* .2023;8(1) : 10.1186/s41073-023-00131-7 10 10.1186/s41073-023-00131-7 37488628 PMC10367367

[2] : The limitations to our understanding of peer review. *Res Integr Peer Rev* .2020;5: 10.1186/s41073-020-00092-1 6 10.1186/s41073-020-00092-1 32368354 PMC7191707

[3] : Blinding Models for Scientific Peer-Review of Biomedical Research Proposals: A Systematic Review. *J Empir Res Hum Res Ethics* .2023;18(4) : 10.1177/15562646231191424 250-262 10.1177/15562646231191424 37526052

[4] : Using simplified peer review processes to fund research: a prospective study. *BMJ Open* .2015;5(7) : 10.1136/bmjopen-2015-008380 e008380 10.1136/bmjopen-2015-008380 26137884 PMC4499682

# Response

**Published:** 2024-08-24

**Authors:** Ivan Buljan

**Affiliations:** Department of Psychology, Faculty of Humanities and Social Sciences in Split, Split, Croatia

Dear reviewers, we are submitting our revised paper and point-by-point responses to your queries. We appreciate your efforts and time invested in our paper.

**Reviewer 3: Approved with reservations**

 The study in hand evaluates whether reviewer comments on research proposals differed after the restructuring of the Marie Sklodowska-Curie Actions (MSCA) Innovative Training Networks (ITN) scoring scheme by eliminating the possibility of scoring using numbers. The authors utilize proposal requests data from 2019 and 2020 and found that linguistic characteristics did not differ after the removal of numerical scores from the specified scoring scheme. The following comments are applicable to the current draft of the manuscript:

 It would be strongly advised that the authors run grammar and typo check, or to obtain language and editing services. The current version of the manuscript has multiple occasions of misspelled words, grammatical errors, and typos. Some examples are figure 1 or 2: the word strengths. First paragraph of discussion: from both. Third paragraph of discussion: the word consensus.

**ANSWER: **Thank you for your comment. We went through manuscript and did grammar check and revised the legends to the figures for clarity.

 Please add an abbreviation section at the end of the manuscript, and make sure that all abbreviations are spelled out at the first encounter throughout the manuscript.

**ANSWER: **Thank you, we have added the explanations in the first encounter now, and the Abbreviations section at the end.

 The current study fills a current gap in the literature as identified by the authors early in the introduction section. This section can probably be restructured for better understanding of the readers, in the following manner: ideally, the first paragraph would discuss general aspects of peer review, therefore, the gap in literature can be moved towards the end of the introduction, after discussing a few aspects, models, shortcomings, etc.

**ANSWER: **Thank you for this suggestion. We cite a recent systematic review on grant review, so we do not consider that it is important to repeat the same information, as we follow the standards on the length of introduction section in biomedical papers.

 Additionally, the authors may provide examples for the quantitative characteristics in the introduction, and/or further elaborate on the effect of quantitative characteristics on reviewers’ evaluation. Providing more information regarding the Marie Skłodowska-Curie Actions (MSCA) would probably help supplement this section and provide a better understanding of the dynamics of this grant. Furthermore, it could have been useful to touch on other possible proposals’ evaluating methods such as scoring and ranking, as can be found in[1]. The last paragraph may be used to highlight further the gap in the literature that the authors aim to bridge with this project, indicating that peer review is highly versatile and a diverse process, yet further focusing on the need to address the general lack of theoretical understanding in multiple aspects of peer review, as can be seen in:  [2], and/or the paucity of experimental research in proposals’ peer review as summarized by [3].

**ANSWER: **Thank you, we cited our previous studies on MSCA grant proposal review, in order to keept this paper more compact and clear. As for other types of evaluating proposals, we cite a recent systematic review on this topics, which has the information suggested by the reviewer.

 The authors may want to further elaborate on the reasons for removing the numerical score from MSCA? Was it for the effect of simplifying the peer review process on reviewers’ engagement and quality of report? as pointed out by  [4]

**ANSWER: **The reason for removal of the numerical scores was an intention to shift focus from numerical scores of the proposals to the text, which represents the feedback on the evaluation process. We have now added this information in the text, along with the reference you suggest.

 The study design is cross sectional but not observational. The latter terminology may be removed.

**ANSWER: **We have removed the term “observational”.

 What is the justification for including data from only 2019 and 2020? Why not expand on the size of data by including more years.

**ANSWER:** Unfortunately, we did not have data from other years on our disposal.

 Further information may be added to the assessment tool, Linguistic Inquiry and Word Count (LWIC) software, such as the validity and reliability, the LWIC scores, possible cutoffs, totals, etc. The authors do provide a reference, but it would just be easier to have it readily accessible while reading.

**ANSWER:** We have uploaded the table with explanations of dimensions, and total scores.  Since we used the summary variables, there is no provided reliability coefficient from the original study (Pennebaker et al, 2015).

 Under the bias heading: What is the role of including the whole cohort of submitted proposals in 2019 and 2020 in eliminating potential sampling bias? Please make it clear for the readers.

**ANSWER:** We aimed to state that we avoided potential problems in the interpretation in case we took only samples from the cohorts in both years.

 In the statistical analysis section: How were the predictors established? Was it based on an analysis or from the literature? It would be advised to clarify this detail.

**ANSWER:** As explained above, since there was a significant number of potential predictors, and this was an exploratory analysis, we decided to conduct variable selection, firstly by univariate testing and then by multivariate testing through logistic regression. The process follows the procedure recommended by :

 Bursac, Z., Gauss, C.H., Williams, D.K. *et al.* Purposeful selection of variables in logistic regression. *Source Code Biol Med* **3**, 17 (2008). https://doi.org/10.1186/1751-0473-3-17

 What we now provide are t-tests for comparison between 2019 and 2020, the initial model and final model for call comparison; and also Kruskal Wallis test due to sample size discrepancy, the initial model, and final model published online publicly. Also, Kruskal Wallis tests were done in R, since JASP program cannot support the size of the analysis.

 The entire procedure is now uploaded on OSF, comparisons, initial and final regression models, as well as descriptive statistics.

 Additionally, please justify using the specified p-value 0.001 in your model, as it appears too stringent.

**ANSWER:** The reason for the P<0.001 was due to the significant number of variables tested, in order to avoid alpha error. However, we now also provide the entire analysis, where the reviewer can see that in logistic regression, all significant variables were significant at P<0.001 level.

 In the results section: The majority of proposals were from the physical science and engineering fields. Did this play a role in the outcome of the study, where no differences were detected before and after? The authors may want to expand on this in the discussion section.

 Additionally, is it possible that COVID19 resultant urgency have affected the review of proposals in 2020?

**ANSWER:** Thank you for your suggestions. Both of them seem plausible, and we added them to the discussion section.

 It would also be useful to further elaborate on the blinding model adopted by the MSCA and how they may have influenced the results.

**ANSWER:** Thank you for this comment. Individual assessment of grant proposal are done independently by each expert, but they are not blinded to the applicant identity. They are blinded to the indentity of other experts, but they meet at the consensus stage. As this part of the evalution did not change, we did not address this in the manuscript as it would not have had an impact on the results.

 In Table 3: How can the average word count be negative? Please provide an explanation.

**ANSWER:** We subtracted results from ESR and IER, and in the table we present only differences. The negative direction only indicates that the values in IER were higher than in ESR.

 It would be great to supplement the discussion section with a few recommendations for future researchers into the topic.

**ANSWER:** Thanks, we added some recommendations in the Discussion section.

 Have the authors considered adjusting for possible confounding factors, such as research domain, etc.? If not , please discuss the possible effects of such confounding factors in the Discussion section.

**ANSWER: **Thank you for your comment. We have not included this in the paragraph on limitation in the Discussion section in previous version but it is included now.

 Thank you for your comments and we are ready to do any additional comments if needed.

 Kind regards,

 Ivan Buljan

# Reviewer report: Reviewer response for version 1

**DOI:** 10.5256/f1000research.153043.r246537

**Published:** 2024-03-12

**Authors:** Sven E. Hug

**Affiliations:** 1 Department of Psychology, University of Zurich, Zürich, north-central Switzerland, 8021, Switzerland; 2 Department of Psychology, Social and Business Psychology, University of Zurich, Zürich, Switzerland

The study examines whether and how the removal of rating scores in grant peer review changes the reviewers’ texts. The analysis is based on data from a funding scheme where the rating scores have been removed at the level of individual reviews (Marie Skłodowska-Curie Actions: Innovative Training Networks, ITN). Data was analyzed using the Linguistic Inquiry and Word Count software (LIWC). The results presented in Figures 1 and 2 show that the removal of rating scores had no effect on the four LIWC categories employed in the study, suggesting that written assessments are not affected by whether reviewers are mandated to assign scores to a proposal or not.

 The study examines a relevant and original research question, uses a unique data set, and employs an analytical approach previously applied to peer review data (LIWC). However, the study needs to be improved in the following respects:

 (1) Please justify why the five panels MAT-PHY-CHE-ENG-ENV were clustered into one “research domain” and why the two panels ECO-SOC were grouped together. The samples do have very different sizes.

 (2) In the section “Statistical analysis”, variables are mentioned that have not been described in the section “Variables analyzed” (e.g., “word count for research excellence weaknesses”). I suggest that all variables used in the study are listed in a table, including the values the variables can take. If the variables are not self-explanatory, please also provide a description of the variable.

 (3) Many analytical categories are available in LIWC. The current study used four. Please explain why these four categories were chosen and why they are appropriate for addressing the research question.

 (4) In the section “Statistical analysis”, it is stated that the variables that were not significant were excluded from further analysis. Does this mean that variables were excluded before conducting regression analyses? Please cite a method paper that justifies this approach. Please state which variables were excluded and indicate their p values? Please report the results of the t-tests and Chi-square tests according to APA standards.

 (5) In the preregistered study design, regression analysis has not been mentioned. Please explain why regressions were computed and how they are related to the research question. Please also explain why the variables were regressed on proposal status (Table 1). Please state which variable was predicted in Table 2 and why it was predicted (does “ITN calls” mean the years of the call?).

 (6) Tables 3 and 4 are extremely difficult to process and understand. Therefore, I cannot follow the conclusions drawn in the last paragraph of page 6. Please describe more clearly how you arrived at your conclusions and/or present the data in a way that is easier to understand.

 (7) “Discussion” and “Conclusions”: Please discuss the implications of your findings for peer review practice. Please suggest what future research could investigate.

 (8) Please upload the R and Jamovi code to OSF so that others can reproduce your analysis.

Is the work clearly and accurately presented and does it cite the current literature?

Yes

If applicable, is the statistical analysis and its interpretation appropriate?

Partly

Are all the source data underlying the results available to ensure full reproducibility?

Yes

Is the study design appropriate and is the work technically sound?

Partly

Are the conclusions drawn adequately supported by the results?

Partly

Are sufficient details of methods and analysis provided to allow replication by others?

No

Reviewer Expertise:

research evaluation, bibliometrics, peer review

I confirm that I have read this submission and believe that I have an appropriate level of expertise to state that I do not consider it to be of an acceptable scientific standard, for reasons outlined above.

# Response

**Published:** 2024-08-24

**Authors:** Ivan Buljan

**Affiliations:** Department of Psychology, Faculty of Humanities and Social Sciences in Split, Split, Croatia

Dear reviewer, we are submitting our revised paper and point-by-point responses to your queries. We appreciate your efforts and time invested in our paper.

**Reviewer 2: Not approved**

**Reviewer comment: **The study examines whether and how the removal of rating scores in grant peer review changes the reviewers’ texts. The analysis is based on data from a funding scheme where the rating scores have been removed at the level of individual reviews (Marie Skłodowska-Curie Actions: Innovative Training Networks, ITN). Data was analyzed using the Linguistic Inquiry and Word Count software (LIWC). The results presented in Figures 1 and 2 show that the removal of rating scores had no effect on the four LIWC categories employed in the study, suggesting that written assessments are not affected by whether reviewers are mandated to assign scores to a proposal or not.

 The study examines a relevant and original research question, uses a unique data set, and employs an analytical approach previously applied to peer review data (LIWC). However, the study needs to be improved in the following respects:

 (1) Please justify why the five panels MAT-PHY-CHE-ENG-ENV were clustered into one “research domain” and why the two panels ECO-SOC were grouped. The samples do have very different sizes.

**Author response:** Thank you for your comment. We used European Research Council Field classification: https://ejoss.euras-edu.org/erc-field-classification/#:~:text=There%20are%2025%20ERC%20panels,Panels%2C%20LS1%E2%80%93LS9). We understand that samples have different sizes, but this approach allowed us to narrow down the number of categories in a logical manner for statistical analysis.

**Reviewer comment: **(2) In the section “Statistical analysis”, variables are mentioned that have not been described in the section “Variables analyzed” (e.g., “word count for research excellence weaknesses”). I suggest that all variables used in the study be listed in a table, including the values the variables can take. If the variables are not self-explanatory, please also describe the variable.

**Author response:** Thank you for your comment, the table with explanations is now provided.

**Table 1**

*Description of LIWC variables used in the study*

Refer Table 1

 For the provided example “word count for research excellence weaknesses”, the variable is word count, but only for a subset of reports that discuss proposal weaknesses.

**Reviewer comment: **(3) Many analytical categories are available in LIWC. The current study used four. Please explain why these four categories were chosen and why they are appropriate for addressing the research question.

**Author response:** We used the four most comprehensive categories, which have been applied in most studies. It is true that by using more specific categories we might find differences, but we would need to apply and compare a vast number of categories to do that. So we opted for a more pragmatic approach in our study. We now state this in the limitations:

 “…Also, we used the most comprehensive categories, which are very broad. LIWC dictionary contains many different categories, and it is possible that by using more specific categories, one may find meaningful differences…”

**Reviewer comment: **(4) In the section “Statistical analysis”, it is stated that the variables that were not significant were excluded from further analysis. Does this mean that variables were excluded before conducting regression analyses? Please cite a method paper that justifies this approach. Please state which variables were excluded and indicate their p values? Please report the results of the t-tests and Chi-square tests according to APA standards.

**Author response:** Yes, since there was a significant number of potential predictors, and this was an exploratory analysis, we decided to conduct variable selection, firstly by univariate testing and then by multivariate testing through logistic regression. The process follows the procedure recommended by :

 Bursac, Z., Gauss, C.H., Williams, D.K. *et al.* Purposeful selection of variables in logistic regression. *Source Code Biol Med* **3**, 17 (2008). https://doi.org/10.1186/1751-0473-3-17

 What we now provide are t-tests for comparison between 2019 and 2020, the initial model and final model for call comparison; and also Kruskal Wallis test due to sample size discrepancy, the initial model, and final model published online publicly. Also, Kruskal Wallis tests were done in R, since JASP program cannot support the size of the analysis.

 The entire procedure is now uploaded on OSF, comparisons, initial and final regression models, as well as descriptive statistics.

**Reviewer comment: **(5) In the preregistered study design, regression analysis has not been mentioned. Please explain why regressions were computed and how they are related to the research question.

**Author response: **Thank you for your observation. Due to the significant number of comparisons, we decided to utilize regression after the preregistration has been done. We now labeled that part in statistical analysis as : “Deviation from the protocol”.

**Reviewer comment: **Please also explain why the variables were regressed on proposal status (Table 1).

**Author response: **We intended to determine whether the linguistic characteristics of IER or CR comments predict the status of the proposal. We now state this in the analysis section.

**Reviewer comment: **Please state which variable was predicted in Table 2 and why it was predicted (does “ITN calls” mean the years of the call?).

**Author response: **We are unsure what the reviewer suggests. The description of the criterion variable is provided below the table. The explanation for ITN is Innovative Training Networks, and it is now added below tables.

**Reviewer comment: **(6) Tables 3 and 4 are extremely difficult to process and understand. Therefore, I cannot follow the conclusions drawn in the last paragraph of page 6. Please describe more clearly how you arrived at your conclusions and/or present the data in a way that is easier to understand.

**Author response: **We apologize to reviewer for the confusion. We added more text to make the results more clear.

**Reviewer comment: **(7) “Discussion” and “Conclusions”: Please discuss the implications of your findings for peer review practice. Please suggest what future research could investigate.

**Author response: **Thank you for your comments, we will add recommendations for future.

**Reviewer comment: **(8) Please upload the R and Jamovi code to OSF so that others can reproduce your analysis.

**Author response: **We have uploaded all analyses, and data, to OSF as JASP files.

 Thank you for your time spent in reviewing our manuscript. 

 Kind regards, 

 Ivan Buljan

# Reviewer report: Reviewer response for version 1

**DOI:** 10.5256/f1000research.153043.r233042

**Published:** 2024-01-12

**Authors:** Mariana Pascual

**Affiliations:** 1 Pontificia Universidad Catolica de Chile, Santiago, Santiago Metropolitan Region, Chile

The manuscript represents a high-quality text with a sound justification and is highly relevant in the discipline. The research focus is clearly established, and the procedures for selecting and analyzing data are explained in detail. 

 The language is formal and precise, and there are no signs of ambiguity that may lead to a lack of clarity. 

 The methodological design is simple and well-described, with explicit references to all the tools and variables selected for data analysis.

 The conclusions are relevant and proportional to the scope of the findings, which are discussed in relation to the corresponding literature.

 Only one specific linguistic resource is mentioned: the use of first-person pronouns. The reader may benefit from more textual data to accompany the broad categories that the article presents. Including specific examples from the data would have been helpful and increased the article's readability. 

 All in all, the manuscript shows a clear command of the topic, clarity of expression, and a sound and appropriate methodological design.

Is the work clearly and accurately presented and does it cite the current literature?

Yes

If applicable, is the statistical analysis and its interpretation appropriate?

Yes

Are all the source data underlying the results available to ensure full reproducibility?

Yes

Is the study design appropriate and is the work technically sound?

Yes

Are the conclusions drawn adequately supported by the results?

Yes

Are sufficient details of methods and analysis provided to allow replication by others?

Yes

Reviewer Expertise:

Linguistics; Discourse studies; evaluative linguistic resources

I confirm that I have read this submission and believe that I have an appropriate level of expertise to confirm that it is of an acceptable scientific standard.

# Response

**Published:** 2024-08-24

**Authors:** Ivan Buljan

**Affiliations:** Department of Psychology, Faculty of Humanities and Social Sciences in Split, Split, Croatia

Thank you for your time and your comments. 

 Kind regards, 

 Ivan Buljan
